# Anatomical Study of the Ulnar Nerve Variations at High Humeral Level and Their Possible Clinical and Diagnostic Implications

**DOI:** 10.1155/2015/378063

**Published:** 2015-07-12

**Authors:** Anitha Guru, Naveen Kumar, Swamy Ravindra Shanthakumar, Jyothsna Patil, Satheesha Nayak Badagabettu, Ashwini Aithal Padur, Venu Madhav Nelluri

**Affiliations:** Department of Anatomy, Melaka Manipal Medical College, Manipal University, Manipal Campus, Manipal, Karnataka 576104, India

## Abstract

*Background*. Descriptive evaluation of nerve variations plays a pivotal role in the usefulness of clinical or surgical practice, as an anatomical variation often sets a risk of nerve palsy syndrome. Ulnar nerve (UN) is one amongst the major nerves involved in neuropathy. In the present anatomical study, variations related to ulnar nerve have been identified and its potential clinical implications discussed. *Materials and Method*. We examined 50 upper limb dissected specimens for possible ulnar nerve variations. Careful observation for any aberrant formation and/or communication in relation to UN has been carried out. *Results*. Four out of 50 limbs (8%) presented with variations related to ulnar nerve. Amongst them, in two cases abnormal communication with neighboring nerve was identified and variation in the formation of UN was noted in remaining two limbs. *Conclusion*. An unusual relation of UN with its neighboring nerves, thus muscles, and its aberrant formation might jeopardize the normal sensori-motor behavior. Knowledge about anatomical variations of the UN is therefore important for the clinicians in understanding the severity of ulnar nerve neuropathy related complications.

## 1. Introduction

Ulnar nerve (UN) is the major branch of the medial cord of brachial plexus, given off in the axilla. It consists of fibers of ventral rami of C8 and T1 spinal nerves. But it often receives additional contribution of C7 fibers through median nerve via its lateral root [1]. In the axilla, UN lies medial to axillary artery and at high humeral level it remains close and medial to brachial artery. In the arm it does not provide any notable branch except for a few vasomotor twigs near blood vessels [1].

An anomalous pattern could be appreciated in the division of trunks and formation of the cords of brachial plexus. However, no such aberrations usually could be noticed in the subsequent arrangement of their terminal branches [2]. Anomalous formations of UN and its unusual communications with neighboring nerves at axilla or at high humeral level are to be noted as it may present a complicating factor during surgical attempts to cause a nerve blockade [3]. An unusual communication between neighboring nerves of the brachial plexus often causes blockade of unexpected areas.

UN variations are consistently located in the origin or course of the distal branches. But, in the available literature describing these variations, it has been noted that much variations in communications between neighboring nerves do exist either in the forearm (most commonly) or in the hand. The present anatomical study aims to identify the variations related to ulnar nerve and to discuss the potential importance of such variations in clinical practice.

## 2. Materials and Methods

The present study was conducted in the Department of Anatomy, Melaka Manipal Medical College (Manipal Campus), Manipal, India. It involved examination of 50 upper limbs of formalin embalmed human cadavers aged between 45 and 60 years. Of the 50 upper limbs 24 were right sided and 26 were left sided. The axillary region of all the limbs was exposed carefully after clearing the entire fascia and to look for the anatomy of ulnar nerve formation from medial cord of brachial plexus. The dissection was further continued towards the anterior compartment of the arm in order to probe any communications of ulnar nerve with neighboring peripheral nerves at high humeral level. Meticulous observation of variant forms and/or abnormal communication if any was made. In the case of persistence of variant communication between neighboring peripheral nerves, the length of the communicating channel has been measured and documented. Relevant photographs of representative specimens were taken and produced herewith.

## 3. Results

**Figure 1:**
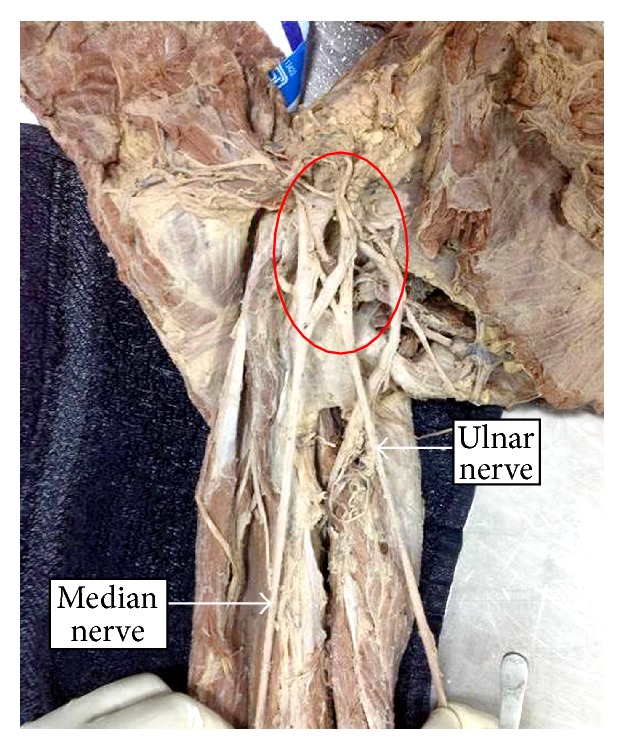
Variant formation of ulnar nerve by the contribution of lateral and medial cords of brachial plexus.

We observed a total of 4 ulnar nerve variations out of 50 upper limbs. It accounted for 8% of incidence cases. All four variations of ulnar nerve were observed in right upper limbs. Aberrant formation of the ulnar nerve with a remarkable contribution from the lateral cord of brachial plexus (Figure 1) was seen unilaterally in two limbs. The ulnar nerve in both the limbs received a contribution from the lateral cord of brachial plexus. The contributing branch passed from lateral to medial side deep to the formation of median nerve and joined the ulnar nerve on the lateral aspect. The formation of ulnar nerve looked similar to formation of median nerve. After its aberrant formation the nerve exhibited a normal course in the arm.

**Figure 2:**
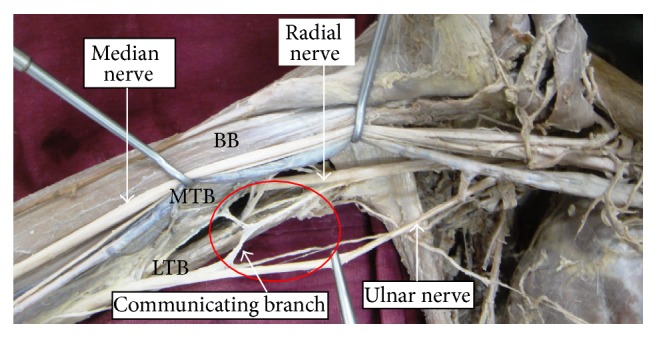
Communication between ulnar and radial nerve. BB: biceps brachii, MTB: medial head of triceps brachii, and LTB: long head of triceps brachii muscle.

**Figure 3:**
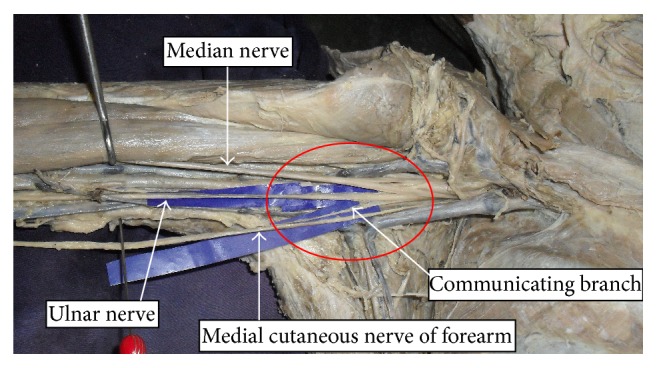
Communication between ulnar and medial cutaneous nerve of forearm.

In the remaining two cases, ulnar nerve had abnormal communications with the neighboring nerves, radial nerve (Figure 2), and medial cutaneous nerve of the forearm nerve (Figure 3). In the case of communication with the radial nerve, the radial nerve prior to coursing the radial groove gave off a communicating branch to the ulnar nerve. In its course, the communicating branch gave off a twig to supply the medial head of triceps brachii (which is visible in the marked area of Figure 2) as a rare occurrence. The communicating branch joined the ulnar nerve before it pierced the medial intermuscular septum. It measured 5.1 cm long.

In case of the communication from medial cutaneous nerve of forearm, the nerve gave off a communicating branch on its medial aspect (Figure 3). The communicating branch sloped medially and joined the ulnar nerve way before the latter pierced the medial intermuscular septum. The communicating branch measured 3.2 cm long.

## 4. Discussion

An effective brachial plexus blockade can be achieved, given that there is possession of thorough knowledge of all possible anatomical variations that can be appreciated in the form of either abnormal origin of its branches or the variant communication between its branches in addition to normal anatomy.

Several studies have reported communications between terminal branches of brachial plexus in the forearm as well as in hand. Different terminology has been allotted to the existence of communication between median and ulnar nerve based on the location of their existence as Riche- Cannieu anastomosis in the palm, Martin-Gruber anastomosis and Marinacci communication in the forearm, and Berrettini anastomosis manifested by the communication between digital branches in the palm. Descriptive features of these communications have been discussed by Dogan et al. [4]. However, in the available literature not much importance has been levied upon the UN variations in terms of both its atypical formation and abnormal communication with the neighboring nerves at the high humeral level or in the axilla. Thorough knowledge on prevalence of variant form of UN is imperative for the clinicians in the diagnostic approaches of sensorimotor impairment syndromes [5].

Variant formation of the UN is not common since there are few reports about it existence. Sachdeva and Singla reported a rare origin of UN from median nerve [6]. In their case, authors noted a bifurcation of the median nerve shortly after its formation into median nerve proper and the UN. Gupta et al. observed a similar type of variation in the formation of UN as we report here, differing slightly wherein there was a contribution from medial root of median nerve [7].

In the present study, we came across with the rare aberrant formation of UN by the remarkable contribution from lateral cord in two of 50 upper limbs. Variations in the origin, course, and distribution of nerves are prone to iatrogenic injuries and entrapment neuropathies [8]. Therefore, during the diagnostic approaches of severity of ulnar neuropathy it is advisable to rule out its aberrant formation.

Unusual communications between the branches of brachial plexus are often seen in medial and lateral cords [9]. Aberrant communication between radial and ulnar nerve is extremely rare at high humeral level though it is often seen on dorsal surface of the hand as evident in available reports in the literature [10, 11]. But there is paucity of reports on their communication at humeral level. The rarest among this was reported by Ajayi et al., as a bilateral ulnar, radial nerve communication at mid humeral level [12]. Fazan et al. reported a prevalence of 30% of cases of UN receiving a communicating branch from musculocutaneous nerve [13]. A limited number of reports about this variation are reported as we understand through the literature survey [7, 14, 15]. However, hardly ever do any reports in the literature confirm the motor distribution of communicating branch. In the current study, the communicating branch between ulnar and radial nerves provided motor innervation to medial head of triceps brachii muscle, which is extremely rare occurrence.

Very rarely might a communication be observed between ulnar nerve and medial cutaneous nerve of forearm. Medial cutaneous nerve of forearm is found to communicate with medial cutaneous nerve of arm [7] and radial nerve [16]. Das and Paul reported a case wherein the ulnar nerve was accompanied by the medial cutaneous nerve of forearm within a common fascial sheath [17]. Knowledge of such variations involving cutaneous nerves is advantageous in nerve grafting and neurophysiological evaluation for diagnosing peripheral nerve lesions.

Diverse opinions of ontogeny of these communications have been put forward by researchers. Probably the simplest cause can be attributed to altered coordination between the mesenchyme responsible for limb muscle and its spinal nerve component which might have led to the formation of aberrant communicating branches [18].

Investigations of peripheral neuropathies are based upon patterns of functional deficits and diagnostic testing. Therefore, an anatomical variation can often lead to confounding patterns of physical and diagnostic findings. According to Ajayi et al., anatomical variant communication between branches of the brachial plexus could obscure the management of complex regional pain syndrome [12].

Ulnar neuropathies are the most frequent causes of nerve injuries as reported by Kroll et al. and they account for a majority with a prevalence of 33%, which is followed by 23% of incidence cases by brachial plexus injuries [19]. Anatomical anomalies are one among the other major risk factors which could be attributed to this cause [6]. Ascertaining the presence of communicating branches may be of importance in the evaluation of inexplicable sensory loss resulting from trauma or surgical intervention in a particular area [20].

Therefore, knowledge on the variant pattern of peripheral nerves is imperative not only for the surgeons, but also for the radiologists during image technology and MRI interpretations and for the anesthesiologists before administering anesthetic agents thus in diagnostic approaches [21]. Damage to communicating roots or nerve may result in weakness with the eventual difficulty in diagnosis [22].

## 5. Conclusion

Awareness of an anatomical variation of UN both in its formation and in abnormal communication at the high humeral level is essential because of the frequency of surgeries performed in these regions for various reasons as well as in diagnostic approaches of management of ulnar neuropathy. Knowledge about anatomical variations of the peripheral nerves is therefore important for the clinicians in understanding the severity of neuropathy related complications.
